# Long gaps between turns are awkward for strangers but not for friends

**DOI:** 10.1098/rstb.2021.0471

**Published:** 2023-04-24

**Authors:** Emma M. Templeton, Luke J. Chang, Elizabeth A. Reynolds, Marie D. Cone LeBeaumont, Thalia Wheatley

**Affiliations:** ^1^ Department of Psychological and Brain Sciences, Dartmouth College, Hanover, NH 03755, USA; ^2^ Santa Fe Institute, Santa Fe, NM 87501, USA

**Keywords:** conversation, gap lengths, long gaps, social connection, turn taking

## Abstract

When people feel connected they tend to respond quickly in conversation, creating short gaps between turns. But are long gaps always a sign that things have gone awry? We analysed the frequency and impact of long gaps (greater than 2 s) in conversations between strangers and between friends. As predicted, long gaps signalled disconnection between strangers. However, long gaps between *friends* marked moments of *increased* connection and friends tended to have more of them. These differences in connection were also perceived by independent raters: only the long gaps between strangers were rated as awkward, and increasingly so the longer they lasted. Finally, we show that, compared to strangers, long gaps between friends include more genuine laughter and are less likely to precede a topic change. This suggests that the gaps of friends may not function as ‘gaps’ at all, but instead allow space for enjoyment and mutual reflection. Together, these findings suggest that the turn-taking dynamics of friends are meaningfully different from those of strangers and may be less bound by social conventions. More broadly, this work illustrates that samples of convenience—pairs of strangers being the modal paradigm for interaction research—may not capture the social dynamics of more familiar relationships.

This article is part of a discussion meeting issue ‘Face2face: advancing the science of social interaction’.

## 1. Introduction

Conversation is a feat of coordination, often characterized by rapid turn-taking. Indeed, the gaps between speech turns tend to be so short (approx. 200 ms [1,2]) that they can only be achieved by predicting what your partner is going to say next [3–5] and planning your response in advance [6–8]. More accurate predictions can facilitate faster response times and shorter gaps between turns. These response times have social consequences [9]. People in conversations with shorter gaps report enjoying their conversations more and feeling more connected to their conversation partners. When people listen to conversations where the gaps have been manipulated to be shorter, they perceive greater connection than people listening to the same conversation where the gaps have been manipulated to be longer. Given that short gaps are an indication that conversation is going well, do long gaps imply that something has gone wrong?

Existing literature strongly suggests that long gaps should be avoided. Long gaps in conversations between strangers are often attributed to poor social skills [10]. Qualitative research asserts that long gaps signal disagreement and sow discord [11–13]. Participants asked to read or listen to conversations that include long gaps report feeling uncomfortable and tend to assume that the people in those conversations feel uncomfortable as well [14,15]. Even watching interactions between a human and a robot that contain long gaps can make people feel awkward [16]. Experimentally lengthening the gap between a request and a response has also been shown to create negative impressions (e.g. reluctance to comply, disagreement [17–20]). Fears of awkward silences may be one reason why people avoid talking to strangers even though doing so is most likely to be enjoyable [21].

In contrast to these findings, a few studies have found that long gaps may not always be problematic. For example, therapists report strategically using silence to encourage reflection and convey empathy [22]. Similarly, long gaps in doctor–patient communication can promote connection and increase patients' feelings of being heard and understood [23]. These findings suggest that, under certain circumstances, long gaps may convey care and reflection rather than awkwardness. Are unproblematic long gaps limited to therapeutic contexts or are they a feature in close relationships more generally?

The goal of the present research is to examine the social implications of long gaps in conversation for both strangers and friends. If long gaps uniformly signal discomfort and awkwardness, then friends may have fewer of them in their conversations compared to strangers. On the other hand, friends may have different types of conversations from strangers [24], many of which may benefit from pauses that promote deep reflection or savouring of inside jokes. This would suggest that long gaps may also be *experienced* differently by friends compared to strangers, which may also be detected by third-party observers. To investigate these questions we examined gaps within unstructured natural conversations between strangers and friends. In Study 1, we tested whether long gaps differ between strangers and friends in terms of frequency and experienced connection. In Study 2, we explored whether the long gaps of strangers and friends are perceived similarly or differently by outside observers.

## 2. Study 1

### (a) Participants

We examined dyadic conversations between strangers and between friends to investigate how long gaps are experienced differently across these two relationship types.

#### (i) Stranger dataset

Participants in the ‘stranger’ dataset participated in exchange for extra credit in their Psychology or Neuroscience courses. Conversation partners were assigned by an experimenter. To ensure that participants did not know each other we asked them ‘How well did you know your study partner before today?’ (0 = not well at all, 50 = moderately well and 100 = extremely well). In order to limit our analyses to true strangers who do not know each other, we excluded 61 dyads where both dyad members indicated a response greater than 0 on this question. The analyses reported in this paper come from 261 stranger dyads. However, note that results are similar with all dyads included.

#### (ii) Friend dataset

All participants in the stranger dataset were invited to participate in the friend dataset. Those who were interested were asked to nominate their close friends to participate with them. Participants in this study had the option of receiving either cash compensation or extra credit in eligible courses. We recorded 65 conversations between dyads of friends.

### (b) Methods

Every conversation session began with two participants having a 10-minute unstructured conversation. Participants were seated across from each other at a cafe table. A webcam attached to a desktop computer across the room captured both participants in profile. After the recording was started, the experimenter turned off the desktop screen so that participants would not be distracted by the recording during their conversation. Participants were told that they could talk about whatever they wanted. After 10 minutes, the experimenter re-entered the room, ending the conversation.

After their conversation, participants were moved to two separate rooms where they privately completed two tasks. They first rated their overall impressions of the conversation via a survey (see electronic supplementary material for all items). They then watched a video recording of their conversation while continuously rating how connected they remembered feeling to their conversation partner at each moment in time. Participants made these ratings by using a computer mouse to move an on-screen slider bar (from 0 = none to 100 = very). The position of the mouse was recorded every 100 milliseconds.

The video recordings of each conversation were transcribed by an external transcription company. Each speech turn in each transcript included the timestamp (in milliseconds) indicating when the speaker started talking and the timestamp when the speaker finished talking. Gap lengths were calculated by subtracting the timestamp at the beginning of a given speech turn from the timestamp at the end of the previous speech turn.

#### (i) Defining a long gap

Although the average gap length in conversation has been well established (approx. 200 ms [1,2,25]), there is no agreed minimum threshold that defines a ‘long’ gap. Here, we considered gaps to be ‘long’ when they lasted more than 2 s (roughly 3 standard deviations from the mean of the distribution; *M* = 248 ms, *s.d.* = 598 ms). Note that gaps here are simply the absence of verbal speech between speakers. Gaps could therefore contain other non-verbal vocalizations or actions.

### (c) Results

### (i) Friends have more long gaps than strangers

**Figure 1.**
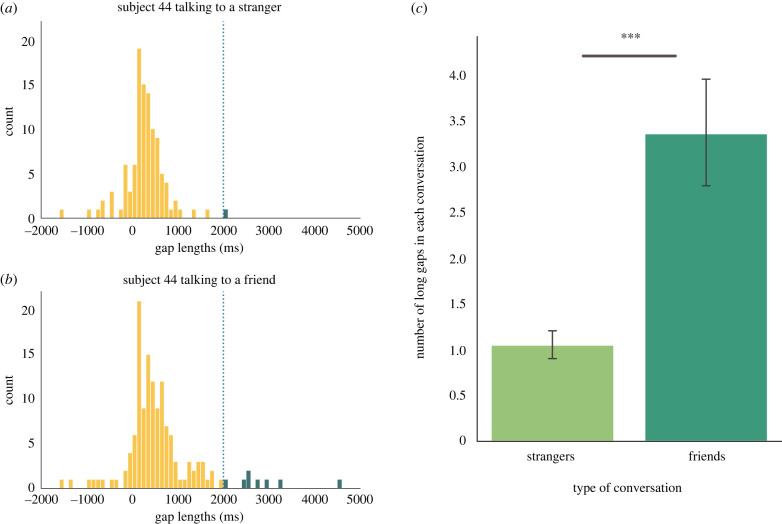
(*a*) Distributions of gap lengths from one stranger conversation. (*b*) Distributions of gap lengths from one friend conversation. All gap lengths over 2000 ms are highlighted in green. Note there are more long gaps when subject 44 talks to their friend compared to a stranger. (*c*) Difference in counts of long gaps for each conversation, split by relationship type. Error bars depict 95% confidence intervals. *** *p* < 0.001. (Online version in colour.)

We first turned to the question of whether long gaps were more prevalent in conversations between friends or strangers. Poisson regression is typically used to model count data. However, we found that our count data were more variable than could accurately be described by a traditional Poisson distribution (i.e. overdispersed: dispersion = 2.95, overdispersion test: *z* = 6.49, *p* < 0.001) and also contained more instances of zeros as a consequence of our long-gap threshold (i.e. zero-inflated; ratio of predicted : observed zeros = 0.76). Therefore, we used a mixed-effects zero-inflated negative binomial regression to predict the number of long gaps based on relationship type (friend or stranger) including subject ID as a random intercept [26]. Because each conversation had a different number of turns, we included the total number of gaps for each conversation as an offset parameter. Results reveal that friends have more long gaps than strangers (*b* = −1.51, *s.e.* = 0.15, *p* < 0.001, figure 1). This finding was robust to varying the threshold for what constitutes a ‘long’ gap (see electronic supplementary material, table S1) and also to the type of statistical model (similar results were found using a negative binomial regression, Poisson regression, and chi-square tests).

#### (ii) Strangers enjoy conversations less when they have more long gaps

We investigated the social consequences of these long gaps by relating counts of long gaps in stranger conversations to participants' own reports of conversation enjoyment. A linear mixed-effect model predicted each participant's rating of how much they enjoyed their conversation based on the number of long gaps in that conversation. We included the total number of gaps for each conversation as a fixed effect covariate and subject ID as a random intercept. Conversations between strangers were rated as more enjoyable when they contained fewer long gaps (*b* = −1.76, *s.e.* = 0.62, *p* = 0.005). Although we were not able to run this analysis in the friend dataset due to their uniformly high and invariant enjoyment ratings, we were able to leverage the continuous connection ratings to examine how connection fluctuated around long gaps between friends as well as strangers.

#### (iii) Changes in connection when entering and exiting long gaps

**Figure 2.**
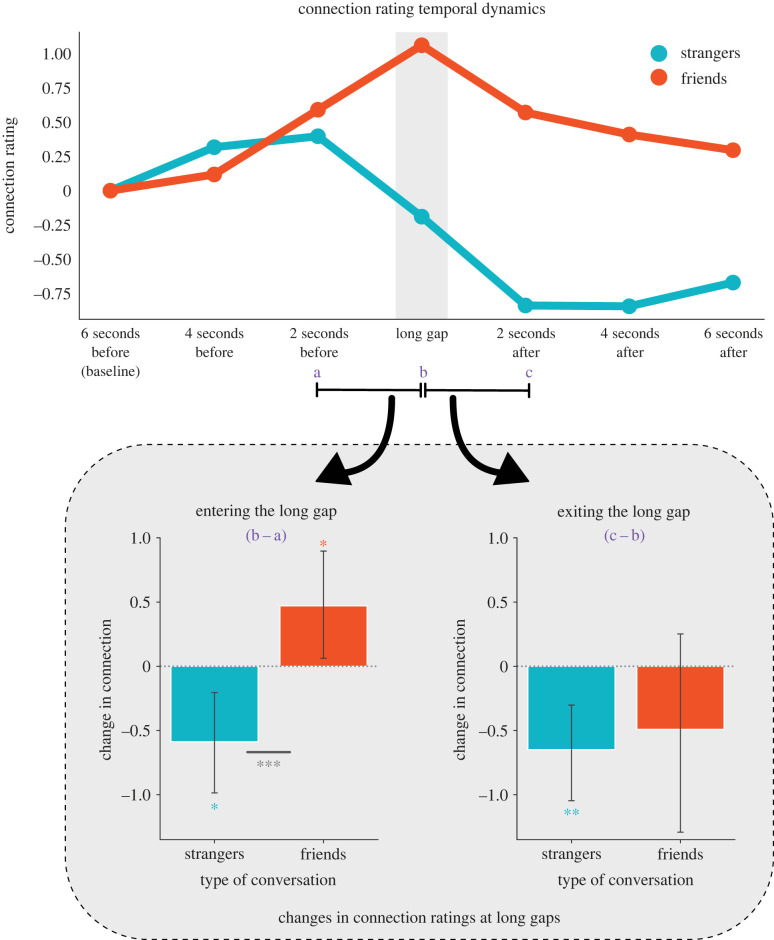
(Top) Depicts the average temporal dynamics of subjective feelings of connection when entering and exiting long gaps, starting at an initial baseline 6 s prior to the gap. Trajectories are plotted separately for strangers and friends. (Bottom) Depicts the changes in connection ratings entering and exiting the long gap separately for friends and strangers. Error bars depict 95% confidence intervals. * *p* < 0.05, ** *p* < 0.01, *** *p* < 0.001. See updated figure with different color intensities. (Online version in colour.)

As expected, friends reported significantly higher average connection in their conversations compared to strangers (*M*_friends_ = 75.55 (*s.d.* = 13.99), *M*_strangers_ = 56.62 (*s.d.* = 19.54), *t(*269.16) = 12.66, *p* < 0.001, *d* = 1.11). But did feelings of connection, for either group, change when entering and exiting long gaps? Because long gaps varied in length, we temporally aligned the data by averaging connection ratings for each long gap into a single time interval. We then computed the average connection ratings at time points before and after long gaps in two second intervals. Mixed effects linear regressions modelled the temporal derivative of ratings entering and exiting long gaps, treating subject ID as a random effect. We found that connection ratings for friends and strangers differed when *entering* a long gap (*b* = 1.03, *s.e.* = 0.35, *p* = 0.004). Specifically, friends' feelings of connection increased going into a long gap (*b* = 0.49, *s.e.* = 0.23, *p* = 0.043), whereas strangers’ ratings decreased (*b* = −0.58, *s.e.* = 0.27, *p* = 0.038). When *exiting* a long gap, ratings decreased significantly for strangers (*b* = −0.67, *s.e.* = 0.22, *p* = 0.004) with no significant difference emerging for friends (*b* = −0.48, *s.e.* = 0.45, *p* = 0.290). Figure 2 shows how connection ratings change over time from the first time point (i.e. 6 s before the long gap), for both relationship types. These findings are robust to varying the threshold for what constitutes a ‘long’ gap (electronic supplementary material, figure S2) and to varying the length of the intervals surrounding the long gap (electronic supplementary material, figure S3). In conversations between strangers, long gaps mark moments of diminishing connection: feelings of connection markedly dip when entering the long gap and remain low afterwards. For friends, long gaps mark moments of heighted connection: feelings of connection start to build, reaching a crescendo at the long gap.

#### (iv) Changes in connection become stronger as gap length gets longer

We next explored whether the changes in connection ratings at long gaps that we observed in the previous set of analyses might be moderated by the duration of the long gap. To test this, we re-ran the same set of analyses described above using gap length to predict the change in connection ratings entering and exiting long gaps. Gap length was log-transformed to account for the exponential distribution of the long gap data (long gaps are defined as being longer than 2 s). For friends, the increase in connection when entering into a long gap was stronger for longer gaps (*b* = 1.66, *s.e.* = 0.77, *p* = 0.031, electronic supplementary material, figure S2). For strangers, increasing gap length was associated with a greater decrease in feelings of connection when exiting the long gap (*b* = −3.83, *s.e.* = 0.71, *p* < 0.001, electronic supplementary material, figure S2). These findings indicate that gap length amplifies the changes in connection ratings observed in friends and strangers in figure 2.

## 3. Study 2

In Study 1, we found evidence that long gaps were more prevalent in friend conversations compared to stranger conversations and that long gaps diminish feelings of connection between strangers while enhancing feelings of connection between friends. In Study 2, we examined whether these differences in felt connection were apparent to outside third-party observers as well. Raters who were blind to the relationship of the conversation partners watched video clips in which long gaps occurred and rated them on a variety of dimensions (e.g. awkwardness, connection, and nonverbal communication).

### (a) Method

Independent raters viewed video clips taken from moments in the conversations that had long gap lengths (i.e. greater than 2 s). After each video clip, the raters rated their impressions of the gap including: dyadic comfort ('How awkward did the gap seem?', 'How connected did the two people seem during the gap?'), nonverbal communication ('Did any laughter occur during the gap?', 'During the gap, did either participant seem to use any gestures with the intent of communicating something?') and topic switches ('How closely related were the two turns surrounding the gap?'). See the electronic supplementary material for the complete list of questions. Raters viewed 100 video clips: 50 from stranger conversations and 50 from friend conversations. Each condition included 10 video clips in each of 5 gap length intervals: 2–2.5 s, 2.5–3 s, 3–3.5 s, 3.5–4 s and greater than 4 s. Raters viewed the video clips in a random order and were not informed that clips came from two different relationship types. Detailed procedures and analysis plan for this rating task were preregistered at https://osf.io/ksnyj.

#### (i) Video clip selection

The final stimulus set consisted of 100 video clips: 50 from each conversation type (stranger and friend). Within each conversation type, we selected 10 video clips with each of the 5 gap length intervals. We used a randomization procedure designed to find clips for each conversation type and interval while also maximizing the number of unique conversations represented in the final stimulus set. This procedure thus minimized the influence of any one converstation on the results.

Each conversation clip contained the full long gap as well as 15 s before the start of the long gap and 15 s after the end of the long gap. These surrounding epochs were included so that raters could consider the context of the long gap. Raters knew that the gap began 15 s into the video clip. The video clips were presented in a Qualtrics survey. Each page of the survey displayed the video clip on top and the set of questions about that clip below. Raters could play the video clip as many times as they wanted to answer the questions about that particular clip. The presentation order of the video clips was randomized for each rater.

#### (ii) Information about raters

Three independent raters viewed and rated all 100 video clips. All of the raters were research assistants approved to be members of the research team by the Dartmouth Committee for the Protection of Human Subjects. None of these research assistants was involved in any of the original studies for which the recordings were made and all were blind to the study hypotheses. The use of research assistants allowed all video-recorded conversations to be rated as opposed to only those with video releases (minimizing potential selection effects). Before completing the rating task, raters viewed and discussed a training set of 24 clips that were not part of the final stimulus set.

Inter-rater reliability scores were computed using Cohen's Kappa for categorical questions (e.g. ‘Did any laughter occur during the gap?’) and intraclass correlation coefficients for continuous questions (e.g. ‘How awkward did the gap seem?’). The majority of questions achieved above moderate inter-rater reliability (see electronic supplementary material, table S2 for inter-rater reliability scores for each of the coded variables).

#### (iii) Models

We used two different approaches to investigate whether raters perceived long gaps differently based on relationship type. For continuous questions (e.g. ‘How awkward did the gap seem?’) we ran separate linear mixed-effects models predicting each rating based on relationship type (friend or stranger), treating rater ID as a random intercept. We report standardized regression coefficients to increase interpretability. For categorical questions (e.g. ‘Did any laughter occur during the gap?’) a ‘consensus response’ was established by taking the modal response across all raters. A chi-square test examined differences in responses by relationship type.

### (b) Results

#### (i) Long gaps in friend conversations are perceived as qualitatively different from long gaps in stranger conversations

**Figure 3.**
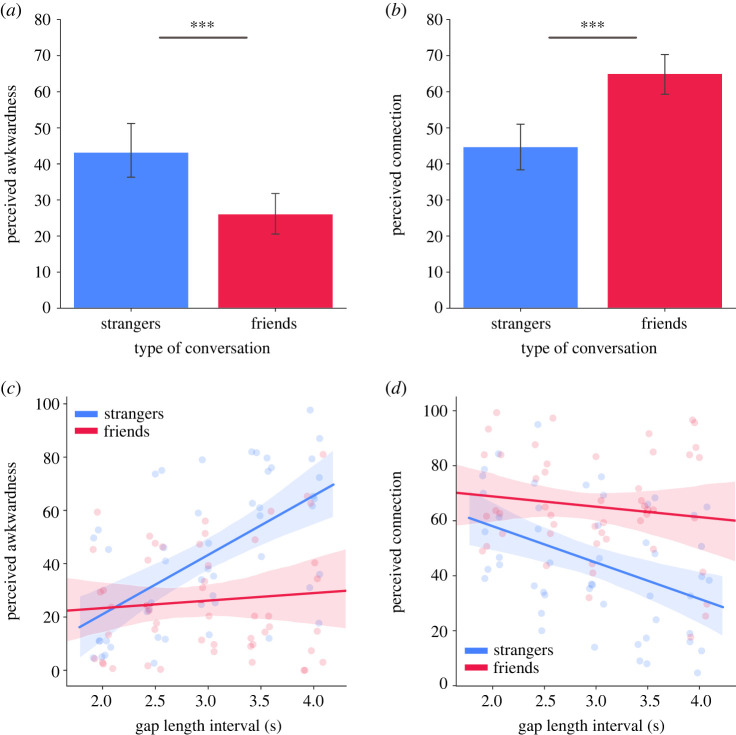
(*a*) Difference in ratings of awkwardness during moments of long gaps in stranger conversations and friend conversations. (*b*) Difference in ratings of connection during moments of long gaps in stranger conversations and friend conversations. (*c*) Effect of gap length interval on ratings of awkwardness split by relationship type (stranger versus friend). (*d*) Effect of gap length interval on ratings of connection split by relationship type. Lines show linear regression model fit. Jitter was applied to show individual data points; however, each data point belongs to one of the five interval bins. All error bars depict 95% confidence intervals. *** *p* < 0.001. (Online version in colour.)

We found that long gaps were rated as less awkward in friend conversations compared to stranger conversations (*b* = 0.59, *s.e.* = 0.11, *p* < 0.001, figure 3*a*) and friends were perceived to be more connected during long gaps relative to strangers (*b* = −0.75, *s.e.* = 0.11, *p* < 0.001, figure 3*b*). This finding appeared to be amplified as a function of the gap length (as indexed by the 5 interval bins). We found a significant interaction between relationship type and gap length on ratings of awkwardness (*b* = 0.47, *s.e.* = 0.10, *p* < 0.001, figure 3*c*), indicating that perceptions of awkwardness *increased* with gap length more for strangers compared to friends. Similarly, a significant interaction between relationship type and gap length on ratings of connection indicates that perceptions of connection *decreased* with gap length more for strangers compared to friends (*b* = −0.25, *s.e.* = 0.10, *p* = 0.017, figure 3*d*).

We also found evidence that long gaps serve different purposes depending on relationship type. For example, strangers were more likely to switch topics after a long gap compared to friends (*b* = −0.29, *s.e.* = 0.11, *p* = 0.011). Whereas a long gap between strangers may create awkwardness and an impetus to change topic, a long gap between friends may serve as a moment to reflect on what was just said. Friends' long gaps were more likely to contain laughter than strangers’ long gaps, *X*^2^ (1, *N* = 100) = 6.05, *p* = 0.014) and when laughter did occur, it was perceived as being more genuine (*b* = −0.48, *s.e.* = 0.19, *p* = 0.011) compared to strangers. This suggests that the laughter of friends is a genuine response to conversational content whereas the laughter of strangers may be an act of politeness to fill time. (See electronic supplementary material, table S3 for the effect of condition on every variable measured.)

## 4. Discussion

For friends and strangers alike, short gaps are a heuristic for connection: the shorter the gap, the more connected people feel to their relationship partner [9]. Here we show that the inverse—the longer the gap, the less connected people feel—is only true for strangers. We found that friends had more instances of long gaps compared to strangers and that long gaps were the site of *increased* connection.

We defined gaps simply in terms of the length of time between verbal speech turns. This definition benefits from being easily computable from an audio file or transcript and therefore facilitates reproducibility and portability to a variety of contexts. It is important to note that the absence of speech in these gaps does not imply the absence of communication. On the contrary, the gaps we investigated here could contain non-verbal vocalizations, gestures, or postural changes. Future research should examine how these different characteristics may affect feelings of connection. It is likely that what happens *within* these gaps is illustrative of the particular meaning or context of that gap and that there may be several meaningful subtypes. For example, previous research has defined a ‘lapse’ as a moment when all participants forgo their turn to speak [27,28]. It is possible that ‘lapses’, as so defined, are gaps that are particularly detrimental for connection. Other gaps may be marked by genuine laughter, with positive consequences for connection. Still other gaps may contain postural changes indicating reflection, and so on.

Findings from Study 2 provide some hints as to how long gaps function differently between friends and strangers. Long gaps between strangers contained less laughter overall and less authentic laughter than long gaps between friends. Long gaps between strangers were also much more likely to be followed by a change in topic, compared to friends (see also electronic supplementary material, figure S4). These findings suggest that long gaps prompted strangers to cast around for something new to say. By contrast, long gaps between friends provided spaces for reacting and reflecting on what was just said. Outside observers also perceived the long gaps of friends as less awkward and more connected compared to the long gaps of strangers—a finding that mirrored the connection ratings of the conversation partners themselves. These findings add critical nuance to previous assertions that long gaps in conversation uniformly signpost trouble [11,17–19,29]. Our results indicate that this is only true in conversations where people are getting to know each other.

The present study further illustrates the importance of expanding interaction research beyond the context of strangers. For most of human history, people have lived in communities in which familiar others are their modal conversation partners [30]. Even in modern, WEIRD [31] cultures in which stranger conversations are not infrequent, people prefer to spend the majority of their social lives with friends and family [32]. In contrast, the modal interaction in communications research is that of strangers. This reliance on stranger dyads may lead to an incomplete, if not distorted, understanding of conversational dynamics. As one example, we show that long gaps are associated with markedly different feelings of connection for friends compared to strangers. Long gaps between strangers are a sign of disconnection, and increasingly so the longer they endure. Long gaps between friends signal heightened social connection, regardless of their duration. It is important to understand how conversational dynamics differ between contexts, how they evolve as relationships grow, and how they may signal relationship health [33]. A fuller understanding of these dynamics will help paint a more accurate picture of what intimacy looks and sounds like.

For people with a shared history, such as close friends, long gaps may simply be times when communication travels ‘inside the head’, as when reflecting on what was just said or mutually savouring past experiences. This can be triggered by a simple word or phrase ('Remember Paris?'). In these instances, a loss of words does not mean a loss of connection or even communication. Because of this, long gaps may not be experienced as gaps at all. The long gaps we remember are instead likely to be ones we enter clumsily and fail to exit gracefully. Such a bias in memory may explain the intuitive yet mistaken assumption that long gaps are uniformly negative. We hope that this work will spur future research that looks more carefully at how features of friends' conversations differ from strangers' and how these differences contribute to their social consequences.

Collectively, these studies suggest that long gaps function differently between strangers and friends. For strangers, long gaps are moments of dead air—awkward silences followed by swift changes in topic. For friends, long gaps may not be accurately described or experienced as ‘gaps’ at all. Though devoid of words, the long gaps of friends appear to be full of meaning, providing natural moments for reflection and expression. These differences between the long gaps of strangers and friends are apparent to outside observers: while the long gaps of strangers are hard to watch, the long gaps of friends telegraph connection. These studies add to a growing literature showing that features of conversation change based on shared history and social context [34–38]. Gaps between turns carry meaningful social consequences, and those consequences change with friendship.

## Data Availability

De-identified data for all studies as well as data analysis scripts are available at: https://doi.org/10.5281/zenodo.7178629 [39]. The data are provided in electronic supplementary material [40].
